# Dose-reduction strategies in whole-body CT for injured patients in the emergency department: a scoping review

**DOI:** 10.1186/s12245-026-01181-6

**Published:** 2026-03-16

**Authors:** Diogo Pereira, Frederic Thevoz, Ana Carolina Rocha, Sabine Schmidt-Kobbe, Pierre-Nicolas Carron, François-Xavier Ageron

**Affiliations:** 1 Emergency Department, Lausanne University Hospital, CHUV, Bugnon 46, Lausanne, CH-1011 Switzerland; 2 Department of Anaesthesia, Lausanne University Hospital, CHUV, Lausanne, Switzerland; 3 Radiology Department, Lausanne University Hospital, CHUV, Lausanne, Switzerland

**Keywords:** Whole-body computed tomography, Low dose, Injury, Polytrauma, Emergency

## Abstract

**Background:**

The use of Whole-Body Computed Tomography (WBCT) is becoming the standard of care for trauma patients as a first approach in the emergency setting. Radiation exposure is one of its major drawbacks. The use of a low-dose WBCT may be the answer to reduce radiation exposure. However, few studies have investigated the impact in clinical practice. This scoping review collected the available evidence comparing dose-reduction strategies in WBCT with the standard of care protocol.

**Methods:**

This study is a scoping review of technical data, the use and effects of low-dose CT in injured patients. Electronic searches were performed using Ovid Medline, Cochrane CENTRAL, Embase, LILACS, Scielo, WPRIM from the date of inception to 5 March 2024. Unpublished trials were identified by searching trial registers, clinicaltrials.gov, the WHO International Clinical Trials Registry Platform (ICTRP), the Cochrane central register of Controlled Trials, conference abstracts and theses.

**Results:**

Twenty-two articles were included, mostly observational and retrospective. Only one prospective randomised trial was reported. The methods used to reduce the dose were the use of an iterative reconstruction technique in 6 studies, a split bolus technique in 8 studies, a different arm positioning in 4 studies, and a single-pass continuous whole-body acquisition technique in 3 studies. The image quality of the low-dose protocols was generally similar or inferior, but the diagnostic capacity remained the same. The lowest effective dose achieved in the different studies for effective dose was 4.8 mSv for a protocol combining iterative reconstruction with a split-bolus.

**Conclusion:**

Across studies, a consistent trend of dose reduction was observed with all strategies, with larger reductions reported for split-bolus protocols. However, direct comparisons were limited by heterogeneity in study designs, scan protocols, and reporting measures of effect, precluding definitive conclusions about the relative superiority of any single approach. Image quality did not seem to be affected by dose reduction. However, there was an increase in missed diagnoses of arterial injury. Direct comparisons were limited by heterogeneity in study designs, scan protocols, and reporting measures of effect, precluding definitive conclusions about the relative superiority of any single approach.

**Supplementary Information:**

The online version contains supplementary material available at 10.1186/s12245-026-01181-6.

## Background

The use of diagnostic imaging has increased steadily in recent years, with growing concerns about its indications and benefits for patient outcomes [1]. Medical imaging is a cornerstone in the accurate diagnosis and treatment of disease. However, it increases the overall cost of healthcare, the environmental impact and could be potentially harmful due to increased radiation exposure associated with an increased risk of cancer, incidental findings, overdiagnosis and the resulting psychological impact.

The aim of a trauma system is to rapidly identify the source of bleeding. In most trauma centres, the overall approach to the immediate assessment and resuscitation of the injured patient follows the Advanced Trauma Life Support (ATLS) protocol. Depending on the physical examination, the anamnestic criteria and the mechanism of injury, clinicians must choose between a selective imaging with either a CT scan focused on a specific anatomical region or a radiography, or a full assessment with a whole-body CT (WBCT). A WBCT typically includes the imaging of the head, neck, chest, abdomen and pelvis. WBCT has become the standard of care in many trauma centres with some systematic reviews showing favourable outcomes in terms of mortality and time to diagnosis [2–7]. However, most of these studies were retrospective and non-randomised. In 2016, the REACT-2 randomised control trial failed to show a statistically significant reduction in mortality with the use of WBCT compared to conventional radiological work-up [8]. Due to methodological concerns and the lack of a clinically significant reduction in the radiation exposure, this trial was unable to recommend the withdrawal of the WBCT.

In recent years, the use of low-dose CT with reduced radiation exposure has increased in both trauma [9, 10] and non-traumatic diseases [11, 12]. This is consistent with the need to establish well asserted guidelines for the overall radiation risk, including low-dose radiation exposure [13, 14], because even low-dose radiation exposure increase the risk of developing cancer, especially in younger patients [15, 16].

Low-dose WBCT appears to be a diverse and heterogeneous practice with neither national nor international standardisation. We aim to explore the available evidence on dose-reduction strategies in whole-body CT in injured patients in the emergency setting by reviewing the evidence on the diagnostic accuracy, clinical utility, clinical setting and radiation dose.

## Methods

### Design and setting of the study

The study is a scoping review assessing the type, the technical characteristics, use and impact of low-dose CT for injured patients. The term “low-dose” is inconsistently defined across studies and does not correspond to a uniform radiation threshold. Most included studies evaluated relative dose reduction compared with local standard protocols rather than predefined low-dose targets. Therefore, the term ‘low-dose’ in this review reflects dose-reduction strategies rather than a standardized low-dose WBCT entity.

### Eligibility criteria

We included all studies published in English and some non-English publications. Studies had to be performed in the emergency setting in an adult or paediatric trauma population. The studies must involve any low-dose CT protocol with or without control group. Publications must report a technical description of the low-dose CT. As we performed a scoping review, we did not exclude any studies based on the reported outcome. We excluded review articles and study protocols. We included some studies that did not report accurate or appropriate dose reduction calculations. These studies using low-dose protocol were included to assess imaging quality, emergency department length of stay or diagnostic accuracy.

### Information source and search strategy

The search strategy combined the free text terms “computed tomography”, “low dose”, “whole body”, “traumatic”, “emergency” and their synonyms, including relevant truncations, MeSH terms and keywords. Electronic searches were performed using Ovid Medline, Cochrane CENTRAL, Embase, LILACS, Scielo, WPRIM from the dates of inception to 05.03.2024. Unpublished trials were also identified by searching trial registers, clinicaltrials.gov, the WHO International Clinical Trials Registry Platform (ICTRP), the Cochrane central register of Controlled Trials, conference abstracts and theses were also made. All non-English publications were translated into English before full-text assessment. The final search strategy for OVID Medline can be found in the supplement file.

### Selection process and data collection

The electronic search results were exported to EndNote, version X8 (PA, USA) and duplicate results were removed. Selection was made by the title followed by the abstract and, finally, full text. Each selection was performed and compared by both reviewers. Data extraction per protocol was performed for each article and the information was exported into an Excel database by each reviewer for comparison. The search query for each database can be found in the appendix. The reviewers (DP, FT) selected and extracted the data. A third reviewer (FXA) assisted in case of disagreement.

Technical data was extracted, when available. If not mentioned in the article, an appendix search was performed. Data extracted were effective dose and dose linear product (DLP) or CT dose index-volume (CTDIvol), to compare radiation dose. No conversion was performed by the reviewers. Data collected included year, country of publication, description of low-dose protocol, efficacy, radiation exposure and any additional details useful in describing low-dose CT.

Computed tomography dose index (CTDIvol), dose length product (DLP) and effective dose (ED) are routinely used to assess and compare the dose received by the patient. ED has been a controversial topic in the past [17] as it was developed for occupational radiation protection and not to be used for individual risk assessment. Some authors have advocated the use of DLP as a comparative measure. The DLP is calculated considering the CTDIvol and the scan range (in cm, not to be confound with the patient’s height). The CTDIvol is a standardised measure of the radiation output of a CT system, determined by the manufacturer measured in a cylindrical acrylic phantom of 16 and 32 cm. To account for the size, organs and body composition of the patient, the ED is calculated by multiplying the DLP by a “k-factor”. The k-factors used are based on a patient of standard height and age [18]. However, the estimation of the effective dose seems to be imperfect with respect of height, age and organ type [19, 20]. Fortunately, manufacturers’ dose calculation software gives a better estimate of organ dose than directly measured organ doses [21, 22].

We reported the results in several tables, including a table listing all studies by year, design and country, a table with technical data, dose reduction and radiation exposure, a table listing the different existing protocols, a table summarising image quality and a table summarising dose reduction by the different protocols used.

We did not assess the study risk of bias as the aim of the study is a scoping review and not a meta-analysis.

This scoping review follows the PRESS Peer Review 2015 search strategy The PRESS 2015 Guideline Assessment Form was filled and reviewed according to the PRESS 2015 Evidence-Based Checklist. The protocol was recorded in the open access platform Figshare [23].

### Statistical analysis

As this is a scoping review, we did not perform a meta-analysis or assess the heterogeneity of the included trials. However, we reported dose reduction and image quality for each study. As the studies used different measures of dose reduction and image quality, we estimated the Cohen’s d as a standardised effect size. Cohen’s d is the difference between the means of the two groups divided by the pooled standard deviation [24].

$$Cohe{n}^{{\prime}}sd=\frac{{\stackrel{-}{\mathrm{X}}}_{1}-{\stackrel{-}{\mathrm{X}}}_{2}}{{Sd}_{pooled}}$$

Where $${Sd}_{pooled}=\sqrt{\frac{\left({n}_{1}-1\right){S}_{1}^{2}+\left({n}_{2}-1\right){S}_{2}^{2}}{{n}_{1}+{n}_{2}-2}}$$

We considered an effect size to be large if Cohen’s d was greater than 0.8. We graphically summarised all Cohen’s d estimates in a forest plot, grouped according to the different low-dose protocols used. Given the methodological heterogeneity across studies, these effect sizes were calculated solely for descriptive purposes. They were used as a visualization tool to facilitate interpretation of heterogeneous data and do not represent pooled, comparative, or inferential estimates. As this is a scoping review, we did not calculate a pooled estimate of dose reduction and image quality.

## Results

**Fig. 1:**
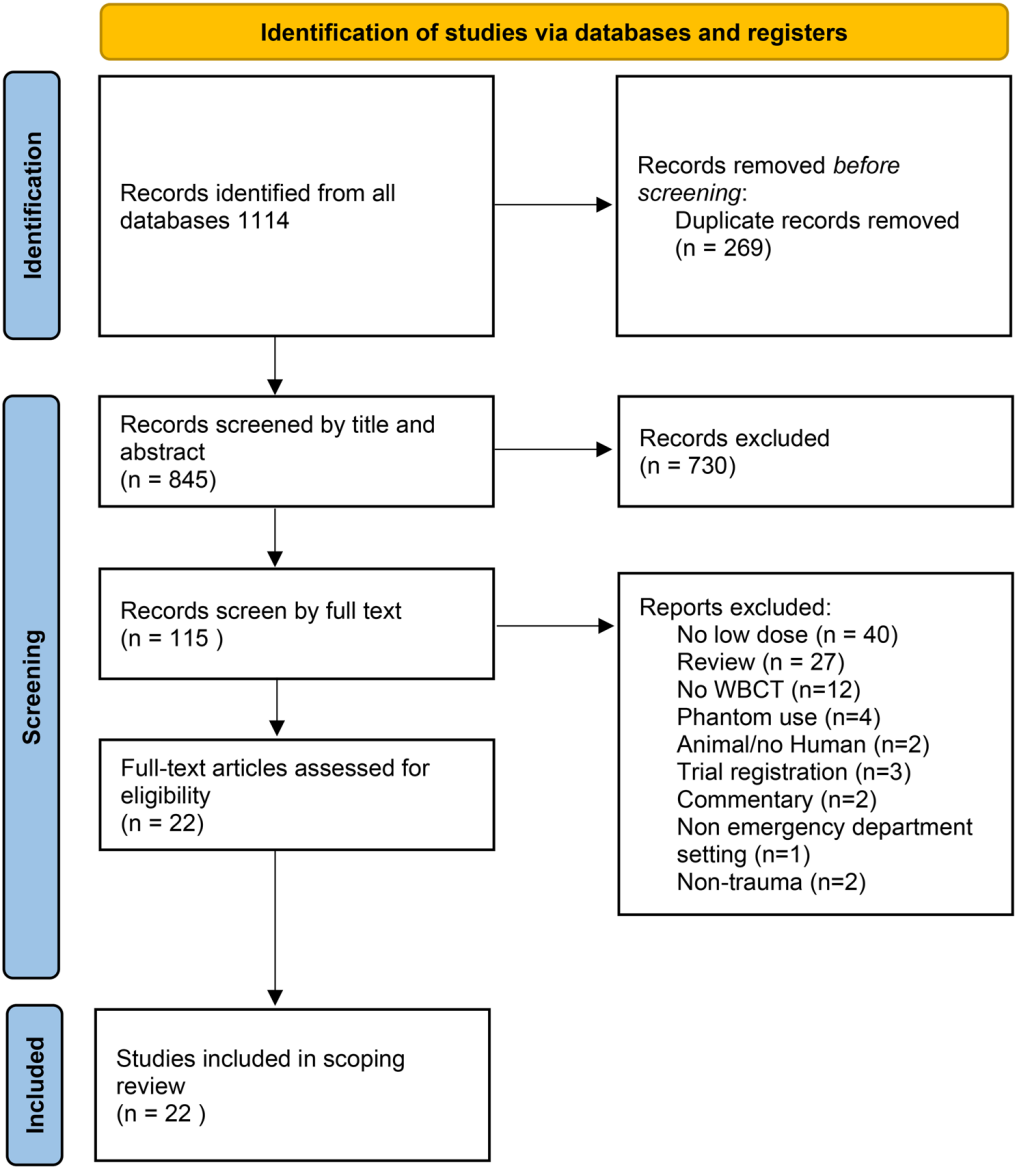
Flow-chart of included articles

We identified 1114 articles (Fig. 1, flow chart of included articles, in accordance to PRISMA [25]). Twenty-two articles met the eligibility criteria (Table 1). All articles included in our review were from Level 1 Trauma Centres, mainly from Europe (77%). Only one study analysed a paediatric population [26]. Eight articles (36%) followed a prospective evaluation [27–34]. The sample size of the studies varied from 18 patients to 971 patients (median 111). Four articles specified the type of trauma, with two including only blunt trauma [29, 35] and two others differentiating between blunt and penetrating [30, 31]. Only four articles used the term low-dose [26, 29, 36, 37].

**Table 1:** Characteristics of the included studies

Author	Title	Country	Year	Language	Design	*N*
**Low-dose protocol used: Iterative reconstruction**						
Grupp et al [27]	Reducing radiation dose in emergency CT scans while maintaining equal image quality: Just a promise or reality for severely injured patients?	Germany	2013	English	Prospective, non-randomized,	18
Kahn, et al [28]	Computed tomography in trauma patients using iterative reconstruction: Reducing radiation exposure without loss of image quality	Germany	2015	English	Prospective, randomised	122
Geyer et al [38]	Dose reduction in 64-row whole-body CT in multiple trauma: an optimized CT protocol with iterative image reconstruction on a gemstone-based scintillator	Germany	2015	English	Retrospective	152
Kahn et al [39]	Quality and Dose Optimized CT Trauma Protocol - Recommendation from a University Level-I Trauma Center	Germany	2017	English	Retrospective	61
Alagic et al [37]	A new low-dose multi-phase trauma CT protocol and its impact on diagnostic assessment and radiation dose in multi-trauma patients	Sweden	2017	English	Retrospective	219
Stengel et al [29]	Association of Low-Dose Whole-Body Computed Tomography With Missed Injury Diagnoses and Radiation Exposure in Patients With Blunt Multiple Trauma	Germany	2020	English	Prospective non-randomized	971
**Low-dose protocol used: Split bolus**						
Yaniv et al [40]	Revised protocol for whole-body CT for multi-trauma patients applying triphasic injection followed by a single-pass scan on a 64-MDCT	Israel	2012	English	Retrospective	82
Beenen et al [30]	Split bolus technique in polytrauma: a prospective study on scan protocols for trauma analysis	Netherlands	2014	English	Prospective	30
Leung et al [41]	Implementation of a split-bolus single-pass CT protocol at a UK major trauma centre to reduce excess radiation dose in trauma pan-CT	UK	2015	English	Retrospective	151
Hakim et al [42]	Trauma whole-body MDCT: an assessment of image quality in conventional dual-phase and modified biphasic injection	UK	2016	English	Retrospective	60
Elmokadem et al [36]	Whole-Body Computed Tomography Using Low-Dose Biphasic Injection Protocol With Adaptive Statistical Iterative Reconstruction V: Assessment of Dose Reduction and Image Quality in Trauma Patients	Egypt	2019	English	Retrospective	100
Ordoñez et al [31]	Implementation of a new single-pass whole-body computed tomography protocol: Is it safe, effective and efficient in patients with severe trauma?	Colombia	2020	English	Prospective, non-randomized	263
Studer et al [43]	Comparison of two whole-body computer tomography protocols for polytrauma patients	Switzerland	2022	English	Retrospective	400
Simma et al [26]	Optimising whole body computed tomography doses for paediatric trauma patients: a Swiss retrospective analysis	Switzerland	2022	English	Retrospective	48
**Low-dose protocol used: Arms position**						
Heyer et al [32]	Prospective randomized trial of a modified standard multislice CT protocol for the evaluation of multiple trauma patients	Germany	2005	German	Prospective	80
Bayer et al [44]	Influence of arm positioning on radiation dose for whole body computed tomography in trauma patients	Germany	2011	English	Retrospective	710
Karlo et al [45]	Whole-body CT in polytrauma patients: effect of arm positioning on thoracic and abdominal image quality	Switzerland	2011	English	Retrospective	150
Harrieder et al [46]	Evaluation of radiation dose in 64-row whole-body CT of multiple injured patients compared to 4-row CT	Germany	2012	German	Retrospective	200
**Low-dose protocol used: Automated Exposure Control (AEC) and arm position**						
Reske et al [47]	Whole-Body CT in Multiple Trauma Patients: Clinically Adapted Usage of Differently Weighted CT Protocols	Germany	2018	English	Retrospective	308
**Low-dose protocol used: Single pass (vs. segmented)**						
Ptak et al [33]	Radiation dose is reduced with a single-pass whole-body multi-detector row CT trauma protocol compared with a conventional segmented method: initial experience	USA	2003	English	Prospective	20
Fanucci et al [34]	Whole body 16-row multislice CT in emergency room: effects of different protocols on scanning time, image quality and radiation exposure	Italy	2006	English	Prospective	46
Sedlic et al [35]	Rapid imaging protocol in trauma: a whole-body dual-source CT scan	Canada	2012	English	Retrospective	67

N : Number of patiens included in each study after exclusion criteria

### Description of protocol and technical data

Technical characteristics for each study are summarised in Table 2. A description of each protocol used is provided in the supplementary file (Table 1). The methods used to reduce the dose were the use of an iterative reconstruction method in six studies [27–29, 37–39], a split bolus technique in eight studies [26, 30, 31, 36, 40–43], a different arm positioning in four studies [32, 44–46] and a single-pass technique in three studies [33–35]. Reske et al. [47] used a combination of different methods with arm positioning and automated exposure control. Studer et al. [43] compared the use of a split-bolus protocol with arm repositioning using a pillow (see the supplementary file for a detailed protocol description).

**Table 2:** Technical data of the studies included

Author	Protocol LD	Other technique	Lowest dose achieved	% relative dose reduction
**Iterative reconstruction -** Post-acquisition software allowing enhancement of image quality by “artificially” removing the noise signal				
U. Grupp et al.	ASIR	Split bolus No mention to arms	17.5mSv (C)	IR 30%: 22% (B) IR 40%: 31% (C)
J. Kahn, et al.	ASiR 40% (except *h*)	Split bolus No mention to arms	12.7mSv (A)	23.4%
L. L. Geyer et al.	ASIR (30% for *h*, 50% for *b*) and Gemstone-based scintillator	Arms crosswise on the trunk	17.2mSv (B)	30%
J. Kahn et al.	ASIR (20–50% full body)	Split bolus No mention to arms	543 mGy (C)	ASIR TAP: 26% (B) ASIR WB: 42% (C)
Z. Alagic et al.	ASIR-V	No reference to arms Increased number of detectors	11.5mSv (B)	13% (if DLP considered. Effective dose NS.)
D. Stengel et al.	iDOSE hybrid iterative reconstruction	Arms on the side	735 mGy (B)	50%
**Split bolus** - an IV contrast splitted in two boluses, in order to enhance both the arterial and venous system in a single acquisition. This technique requires only one image acquisition, instead of two (one for arterial phase and one for venous phase).				
G. Yaniv et al.	Split bolus	No mention to arms/AEC	12.4mSv (B)	32%
L. F. Beenen et al.	Split bolus	Care Dose 4D (AEC) Arms alongside head	1125mGy	Not reported
V. Leung et al.	Split bolus	Care dose and Care kV (AEC) No reference to arms	5.5mSv (B)	49%
W. Hakim et al.	Split bolus	No reference to arms neither AEC	8.86mSV (C)	46% (B) 68% (C)
A. H. Elmokadem et al.	Split bolus	ASIR-V 50% No reference to arms	18.0mSv (B)	36%
C. A. Ordoñez et al.	Split bolus	No reference to arms neither AEC	15mSv	No group control
S. Studer et al.	Split bolus	Arms on pillow ventrally No reference to AEC	15mSv(B)	35%
L. Simma et al.	Split bolus	Care dose 4D + SAFIRE iterative reconstruction	4.8mSv (B)	77%
**Arms position -** reduce the radiation delivered over the body by removing the arms from the field. Arms are moved throughout the acquisition process				
C. M. Heyer et al.	Without gantry angulation and with arms alongside body	No reference to AEC	10,2mSv (B)	20%
J. Bayer et al.	Arms up	CareDOSE	19.18mSv(B)	22%
C. Karlo et al.	Arms up	Care 4D	16.1mSv(A)	24% (arms up) Pillow : increase in radiation
A. Harrieder et al.	Arms crossed on abdomen on 64, upwards for TAP on 4-slice	Care dose 4D (xy plane on 4 row and also z plane on 64)	22.4 mSv (B)	17%
**Automated Exposure Control (AEC) and arms position -** automatically interruption of exposure once a preset dose range is reached and based on the relative size of the patient over his length and the various tissue absorption				
S. U. Reske et al.	Arms up Exposure 3D (AEC)		21mSv (C)	AEC: 34% (B) Arm position: 45% (C)
**Single pass (vs. segmented) -** prevents multiple exposure with the superposition of adjacent body segments and allows dose reduction compared to CT segmented				
T. Ptak et al.	Single pass	Arms are placed in foam ramps at 25–30º from the table	2671mGy(A)	17%
E. Fanucci et al.	Single pass	No reference to AEC or split bolus	2671mGy (A)	17%
A. Sedlic et al.	Single pass	Care Dose 4D (AEC)	24.7mSv (B)	25%

Lowest dose achieved : median effective dose of lowest dose protocol, in mSv. If not available, presented DLP in mGy. Other technique: not used as a comparing variable between groups. Protocol that achieved minimum dose showed between parentheses. Iterative reconstruction technique: ASIR, ASIR V, iDOSE. AEC technique: CARE Dose, Care 4 Dose

Iterative reconstruction corresponds to a post-acquisition software with multiple reconstruction, which improves image quality and allows dose optimisation compared with filtered back projection with a single reconstruction: The latter is today no longer used in the clinical routine. Studies reported on different iterative reconstruction algorithms developed by the manufacturers from the first developed versions, i.e. AsiR (GE Healthcare) and iDose (Philips Healthcare), mainly focused on iterative noise reduction techniques to more advanced hybrid algorithms combining reconstruction and noise reduction, i.e. SAFIRE (Siemens Healthineers), SURE Exposure (Toshiba) and ASIR-V (GE Healthcare). Most of the studies used a 64-row system with a 120 kV tube current and variable amperage depending on the body segment. Only one article varied the parameters according to the body weight [29]. Technical details are summarised in supplementary file 1.

### Dose reduction

**Fig. 2:**
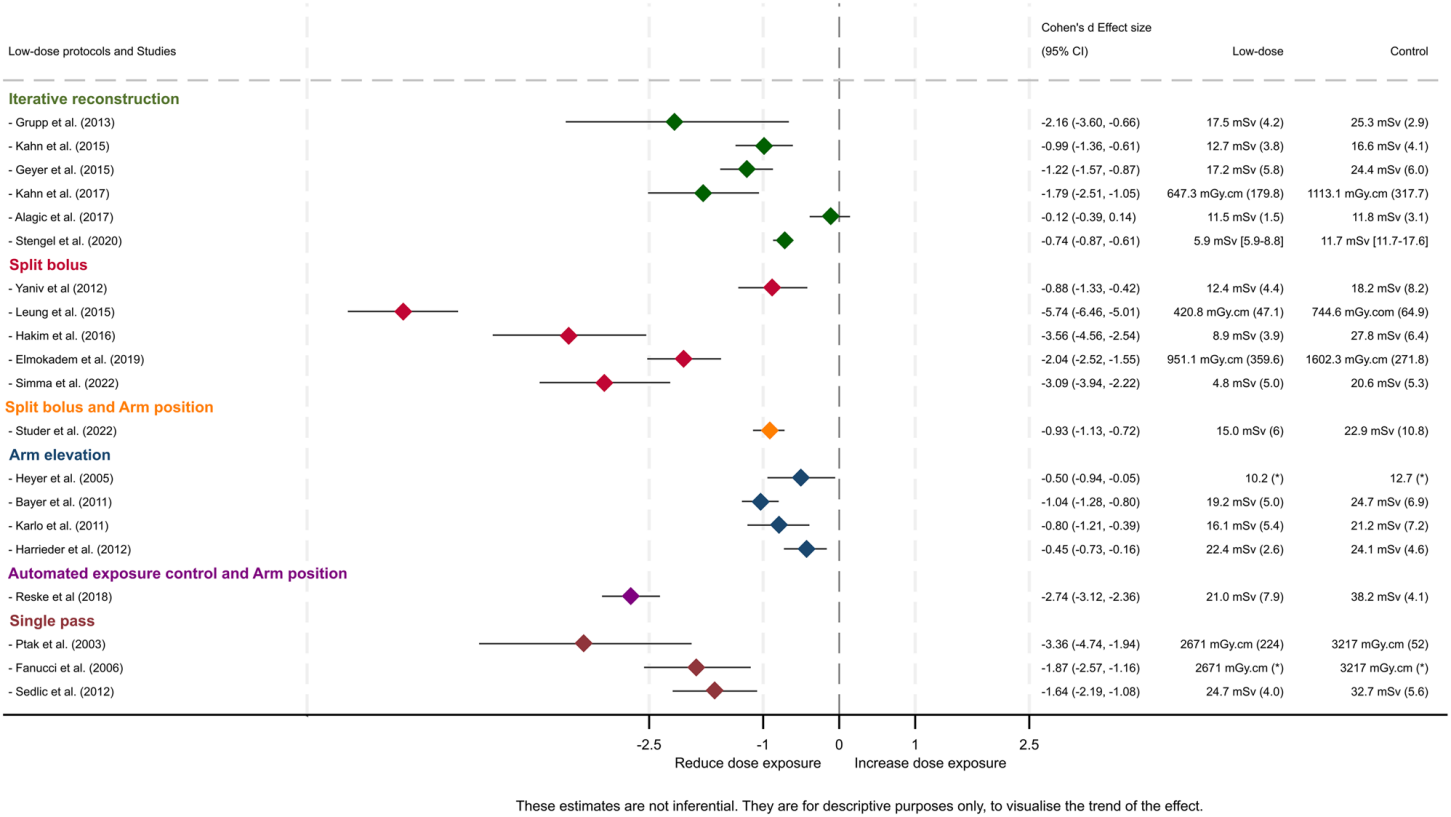
Effect size in exposure dose reduction by different low-dose protocols

Seventeen studies reported the effective dose, calculated with the DLP x coefficient factor from different sources [26–28, 31, 32, 35–38, 40–47]. Five of these did not report the coefficient used [31, 32, 37, 41, 47] (supplementary file, Table 2). Five studies reported only the DLP [29, 31, 33, 34, 39, 41]. All methods allowed for a dose reduction (Fig. 2). Higher average dose reductions were seen with the split bolus technique, the AEC and the single pass. Only Alagic et al. [37] showed no significant difference in effective dose for iterative reconstruction. Beenen et al. [30] who did not assess the effective dose, found no statistical difference in radiation exposure (same DLP) using a split-bolus technique. Most of the studies assessing arms position reported lower radiation exposure when position the arms were positioned above the head during acquisition of thoraco-abdominal images (after unenhanced cranial and cervical imaging) [44, 45, 47], except for Heyer et al. [32] who showed lower exposure dose and examination time when the arms were positioned alongside the body without gantry angulation. The lowest effective dose achieved in the various studies for effective dose was 4.8mSv [26] for a protocol combining a “Care Dose 4D” and a “SAFIRE” iterative reconstruction with a split-bolus protocol [26].

### Image quality

**Fig. 3:**
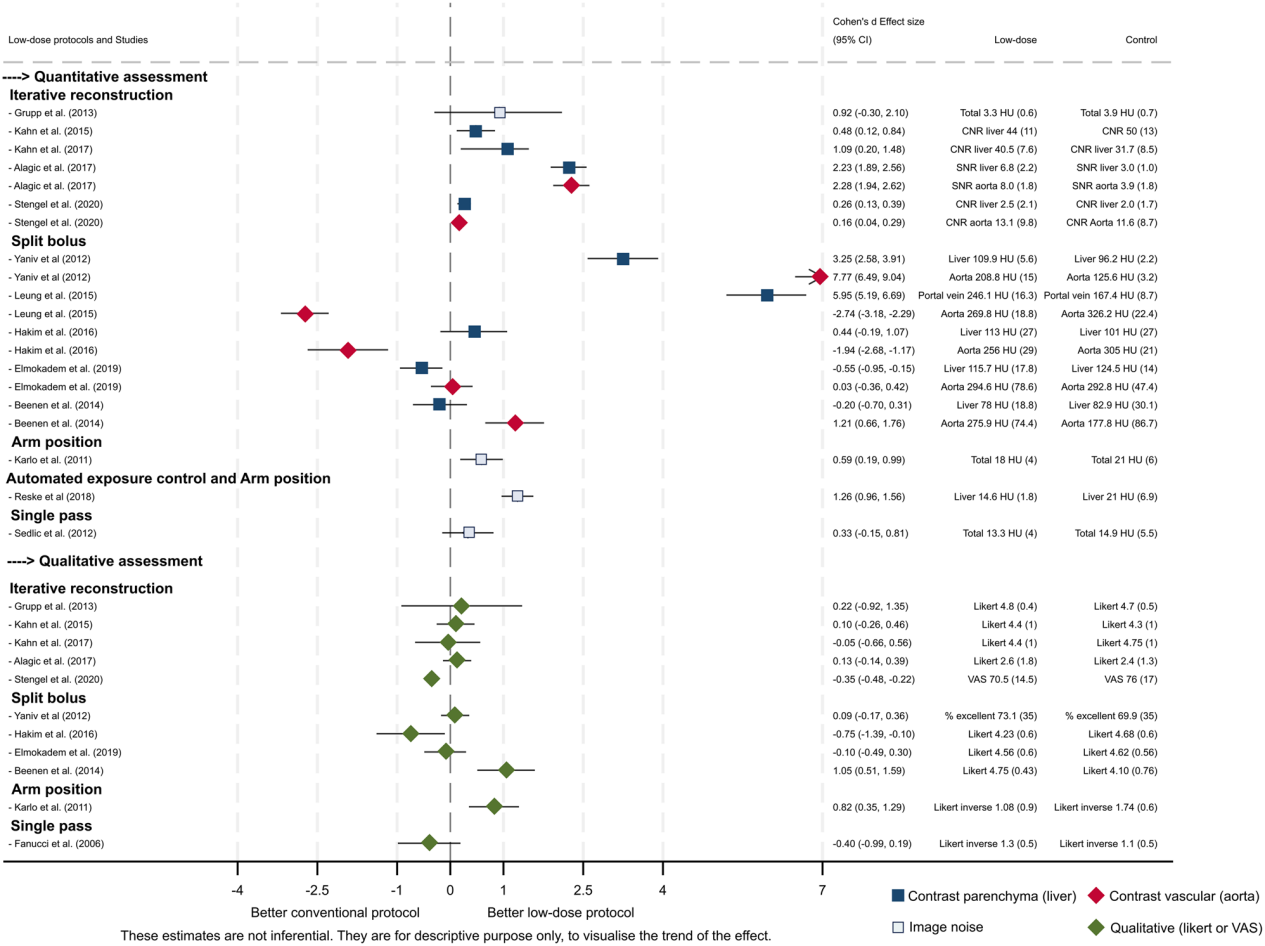
Effect size in image quality by different low-dose protocols

Figure 3 summarises the assessment of image quality in terms of quantitative and qualitative assessment. Studies reported quantitative assessment by using contrast to noise ratio (CNR), signal to noise ratio (SNR), CT attenuation value in Hounsfield units (HU) in different regions of interest (aorta, liver or various organs, bone) or as image noise. When noise was evaluated, lower values indicated better image quality, whereas for organ attenuation or enhancement, higher values indicated better image quality. Of the studies assessing iterative reconstruction, three studies reported better image quality with a higher contrast-to-noise ratio [28, 29, 39] and one study reported no difference [27] (supplementary file, Table 4). The split bolus technique showed a similar image quality. Arms elevation showed better image quality than the arms close to the body. Qualitative assessment reported the radiologist’s subjective assessment of image quality using either the Likert scale or a visual analogue scale. All studies reported a similar qualitative assessment for conventional or low-dose protocols (Fig. 3).

### Accuracy and impact

Regarding mortality, four articles reported data, with no significant difference between low-dose and conventional protocols [30–32, 35].

Regarding WBCT time, one study assessing iterative reconstruction showed a significant longer scan completion time, 28 min versus 21 min; *P* < 0.001 [37]. Two studies assessing split bolus reported no difference in scan time [36, 40] and two other studies showed a decrease of the scan time with split bolus [8, 43]. For arm position, two studies reported longer time for arm repositioning [32, 47] and two studies reported no difference [44, 45]. Studies assessing single-pass showed a decrease in the scan time compared to segmented acquisition [34, 35].

Regarding diagnostic performance, only two articles reported the sensitivity and the specificity of the low-dose protocol. Stengel et al. [29] showed a low sensitivity for haemothorax (40%), hollow visceral tears (0%), haemoperitoneum (29%) and retroperitoneal bleeding (33%) for the low-dose protocol with iterative reconstruction. These sensitivities were estimated for the first results reported to the trauma team immediately after the CT scan realisation. They also reported similar low sensitivities for the standard dose protocol. They did not show an increased risk of missed diagnosis with low-dose protocol compared with the standard-dose. Alagic et al. [37] showed a sensitivity of 67% for the low-dose technique with ASIR-V compared with 100% with conventional protocol. They did not report a P value or a confidence interval. They reported missed diagnoses in arterial injury, 4 out 12 arterial injuries (33%) leading to additional CT angiography in nine patients (8%). They reported no missed diagnoses in non-arterial injuries. In a study without a control group, Ordonez et al. [31] reported 67 patients (25%) requiring additional selective CT in nonsurgical patients, mainly in the brain.

### Other considerations

Only one study assessed different protocols according to haemodynamic stability [47]. Injured patients with haemodynamic instability had a higher Injury Severity Score (ISS) and benefited from a low-dose protocol with automated exposure control. Injured patients without haemodynamic instability had a lower ISS and benefited from a low-dose protocol with automated exposure control and a pause between the head/neck and body for arms repositioned over the head. Both protocols delivered a lower effective dose than the conventional protocol. The scan time was shorter for the former protocol (4.1 min versus 7.7 min, *p* < 0.05), but the effective dose was higher (35 mSv versus 28 mSv).

Ordonnez et al. [31] assessed a single-pass low-dose protocol in different clinical situations : blunt trauma with haemodynamic instability, blunt trauma with haemodynamic stability and penetrating trauma. The authors found no difference in the expected standardised mortality between the 3 groups.

## Discussion

### Main findings

All studies were performed in level 1 trauma centre. We identified several methods to reduce radiation dose: (1) Iterative reconstruction, (2) Split bolus, (3) Automated Exposure Control, (4) Arms position and (5) Single pass. Across the included studies, a consistent trend emerged of dose reduction associated with all strategies. Variability in the magnitude of effect size was observed between studies with a trend of larger reductions in dose metrics for split bolus compared to others. However, direct comparisons were limited by heterogeneity in study designs, scan protocols, and reporting measures of effect, precluding definitive conclusions about the relative superiority of any single approach. Although quantitative and qualitative image quality was generally comparable between protocols, only two studies investigated diagnostic accuracy, and an increased rate of missed arterial injuries was reported. Most of the studies were observational and retrospective. Only one study was a prospective randomised trial. A meta-analysis was not possible because of methodological weaknesses in the studies.

### Interpretation of the results

Low-dose WBCT has the potential to become an important tool for rapid decision making in emergency patients. To minimise radiation exposure as low as possible, iterative reconstruction and split bolus have been the most commonly used techniques. Iterative reconstruction itself does not reduce radiation exposure once acquisition is completed. Rather, it enables lowering of acquisition parameters while maintaining acceptable image quality. However, iterative reconstruction has now been implemented as the method of choice for CT image reconstruction for any type of CT examination, while the old technique, based on filtered back projection, is no longer used. In addition, other types of image reconstruction based on deep learning methods will allow further reductions in patient dose exposure, although we did not find any publications on these newer techniques, in an emergency setting [48, 49].

The net benefit of low-dose WBCT lies between the reduced risk of a lifetime attributable risk of cancer and the risk of a missed injury diagnosis. Reassuringly, even if an additional targeted CT is required, the total dose used should still be lower than conventional protocols. In addition, the use of an additional image after low-dose WBCT will be reserved for patients with a high pre-test probability, where the diagnostic benefit outweighs the radiation-associated risk. However, performing an additional CT acquisition on injured patients is time-consuming and disrupts the smooth flow of patients through emergency departments, which are often overloaded.

Dose reduction in split-bolus protocol results from reducing the number of acquisition phases rather than automatically lowering exposure parameters. However, active haemorrhage and pseudoaneurysms are more difficult to detect with biphasic CT and for this reason Hakim and al [42] decided to continue with the convention of two-phase arterial and portal phase WBCT in all penetrating injuries and patients with major injuries. Another disadvantage of the split-bolus protocol is the higher contrast medium dose required [34] which raises the question of the renal effects of iodinated contrast. Although this is less of a concern in younger adults, trauma increases in the elderly, making the split-bolus protocol a concern in this vulnerable population.

Regarding the assessment of image quality, both quantitative and qualitative studies were in favour of the low dose or showed no difference between the different protocols. These results should be interpreted with caution, as only two articles investigated diagnostic accuracyStengel et al. [29] using an AEC for the low dose protocol gave a quantitative image result in favour of the low dose protocol and no differences in missed injuries between groups. However, they reported a high proportion of missed injuries in both groups on the first reading of the CT scan that were immediately reported to the trauma team. These missed diagnoses should alert clinicians to the unreliability of immediate and early reporting.

Time is a key issue in the emergency setting. Few of the articles included in this review reported the time variables. While the reduction in radiation dose may be beneficial for the long-term exposure, the time spent waiting for the radiologist’s report may be detrimental. This is particularly important when using automated exposure, as the version used affects the time taken to reconstruct the image [50]. Alegic et al. [37], who compared a 258-slice multiphase with AsiR-V to a 64-slice single phase, also reported the time from completion of the examination to the preliminary written radiology report, and found no statistically significant difference was found with a waiting time of 46–48 min. However, the time from registration to examination was increased (*p* < 0.05), raising the question of whether newer, more sophisticated imaging algorithms may increase the time to diagnosis, although other confounding factors may be involved. In the split-bolus group, Elmokadem et al. [36] reported the duration of the examination, which was similar in both groups. Studer [43] and Beenen [30] reported a faster CT without data on the clinical impact.

### Clinical implications

Reduced-dose techniques seem appropriate to reduce patient exposure. Given the generally preserved image quality reported in several studies, dose-reduction protocols may be considered in selected clinical situations. However, clinical assessment and bleeding risk stratification on admission must be performed and taken into account in the decision-making process. In patients with a high risk of bleeding, low-dose protocols should be avoided. On the other hand, in patients with a low risk of bleeding, the use of low-dose WBCT to detect significant injury may be appropriate.

### Strengths and limitations

We performed a rigorous literature search using a precise search equation in different databases to avoid any selection bias. However, some trials may have been missed because of an unclear definition of low-dose protocol. We tried to include trials even if they did not mention the term ‘low-dose’ but referred to dose reduction.

Furthermore, the included studies reported heterogeneously on the different parameters of the dose received and the protocol used. It was not always possible to determine exactly what was done, which led to significant information bias. In this review, the comparison of exposure doses by protocol seemed unreliable because the method used by each author was not standardised. Although DLP seems to be better for comparing protocols [20], 77% of the articles only reported the effective dose, which cannot be compared between articles because of differences in methods [51, 52]. In addition, 29% of the studies included in this review did not explain their method of calculating the effective dose. Technical parameters were poorly reported. Many studies did not report complete information such as beam collimation or field of view, which have been shown to be important in determining the dose delivered to target organs due to scatter [21]. This information bias in dose reporting is a major limitation in assessing and comparing the risk/benefit of a low-dose WBCT.

Moreover, the impact of incidental findings was not measured, nor was the long-term impact.

Additionally, the time taken to identify the source of bleeding could be considered in clinical decision making and is lacking in most of the included studies.

Most included studies were retrospective and single-centre, and only one randomised controlled trial was identified. As a result, the overall level of evidence remains limited, and causal conclusions cannot be drawn.

Only one study focused on a paediatric population, despite the particular importance of radiation exposure in children due to their increased lifetime radiation sensitivity. This markedly limits the generalisability of our findings to paediatric trauma patients.

Although image quality was frequently assessed, only two studies evaluated diagnostic accuracy, and clinically relevant outcomes such as missed injuries were rarely reported. Therefore, preserved image quality should not be interpreted as equivalent diagnostic safety.

Even if we use the Cohen’d estimate as a standardised method of reporting the effect size, the use of heterogenous measure of the radiation dose received limit the clinical meaning of the result. These results are presented solely to illustrate the various existing dose-reduction strategies and summarise their tendency to reduce, or not, the radiation received by patients.

Finally, all the studies included in this review were performed in level 1 trauma centres. The generalisability of the results may be limited. Radiologists working in these specialised centres have more experience in the field of trauma and are better able to suspect and identify specific injuries. We cannot generalise these results to all hospitals that receive trauma patients.

## Conclusion

Low-dose whole-body computed tomography in the emergency setting is a promising tool for clinicians but needs further technical improvement because diagnostic image quality cannot always be maintained. In patients with severe trauma, low-dose WBCT should not be the first-line imaging test for all patients but should be incorporated into a decision-making process that includes bleeding risk stratification.

We have described several methods used to reduce radiation exposure to the lowest level reasonably achievable. A standardised approach to WBCT protocols is needed to allow meaningful comparisons between studies. Future research should focus on clinical outcomes, including mortality rates, time to diagnosis, and the impact of complications such as misdiagnosis. A randomised trial with a large sample size assessing a patient-centred outcome is still needed.

## Supplementary Information

Below is the link to the electronic supplementary material.

Supplementary Material 1

## Data Availability

No datasets were generated or analysed during the current study.

## Abbreviations

AEC: Automated exposure control
AsiR: Adaptive statistical iterative reconstruction
CNR: Contrast to noise ratio
CTDIvol: Computed tomography dose index
DLP: Dose-length product
ED: Effective dose
HU: CT attenuation value in Hounsfield unites
ISS: Injury severity score
SNR: Signal to noise ratio
WBCT: Whole body computer tomography
