# Supplementary material for: Dose-reduction strategies in whole-body CT for injured patients in the emergency department: a scoping review
Source: Int J Emerg Med. 2026 Mar 16;19:121. doi: 10.1186/s12245-026-01181-6 (PMC13107676; doi:10.1186/s12245-026-01181-6)
Supplement: Supplementary file 1 — Supplementary Material 1 [file 12245_2026_1181_MOESM1_ESM.docx]

| **Table 1 – Protocols used** | | | |
| --- | --- | --- | --- |
| **AUTHOR** | **PROTOCOL A** | **PROTOCOL B** | **PROTOCOL C** |
| **Iterative reconstruction** | | | |
| U. Grupp et al.. | Unenhanced H, enhanced WB, with FBP | Unenhanced H, enhanced WB, with ASIR 30% | Unenhanced H, enhanced WB, with ASIR 40% |
| J. Kahn, U et al. | Unenhanced H with FBP, enhanced ASiR 40% for body | Unenhanced H FBP, enhanced NTAP (all FBP) | - |
| L. L. Geyer et al. | Unenhanced head, enhanced NT, enhanced AP, use of FBP | Unenhanced head, enhanced NT, enhanced AP, use of ASIR and gemstone-based scintillator | - |
| J. Kahn et al. | Filtered back-projection (FBP), single acquisition arterial and venous phase WB | FBP for the head, Adaptive statistical iterative reconstruction (ASIR) Wbody (between 30 and 50%), single acquisition arterial and venous phase | ASIR full body (between 20 and 50%), single acquisition arterial and venous phase. Tube voltage reduced to 120kv |
| Z. Alagic et al. | 258-slice MDCT: unenhanced HN, arterial WBCT, venous abdomen with ASIR-V | 64-slice MDCT: unenhanced HN, venous TAP only | - |
| D. Stengel et al. | Unenhanced H, enhanced WB | ASIR, unenhanced H, enhanced WB | - |
| **Split bolus** | | | |
| G. Yaniv et al | Unenhanced examination of the head and neck and upper abdomen followed by 2 phases : chest angiography and porto-venous abdomen and pelvis | Unenhanced examination of the head and neck and upper abdomen followed by a single-phase carniocaudal spiral acquisition fof the chest, abdomen and pelvis | - |
| L. F. Beenen et al. | Conventional unenhanced HN (arms alongside body), enhanced portovenous TAP (arms alongside head) | Brain than one-volume contrast: unenhanced HN, enhanced one contrast volume multiphase WBCT, arms always alongside body | Conventional unenhanced HN (arms alongside body), Split-bolus enhanced TAP with arms alongside head |
| V. Leung et al. | Three phases: unenhanced HN, arterial TAP, venous AP | Split-bolus: unenhanced H, split-bolus NTAP | - |
| W. Hakim et al. | Defined as conventional whole-body MDCT, not specified. Single injection 2-phase acquisition - arterial 30s and PV 60s. | WBCT (not specified), Biphasic injection (65mL - 43s - 65mL - scan), single spiral acquisition | Same as B, further 9s delay before acquisition. Biphasic injection (65mL - 43s - 65mL - 9s delay - scan |
| A. H. Elmokadem et al. | Triphasic protocol: Unenhanced HN, arterial phase TAP, venous phase AP / ASIR-V 0- 50% | Biphasic injection protocol: unenhanced HN followed by a 1 step arteriovenous acquisition of the TAP / ASIR-V 0- 50% | - |
| C. A. Ordoñez et al | New WBCT protocol : 2 phases : simple acquisition phase of skull. Second phase of contrast administration: neck, thorax and abdomen IV contrast : Iodinated, non-ionic hypo-osmollar (370mg/mL) with sequential contrast bolus resulting a single acquisition combining arterial and portal venous phases. Blunt stable vs Blunt instable vs Penetrating accident | | |
| S Studer et al. | Three-phased CT in which a repositioning of the arms from the side of the torso to above the head between phases two (angiography head and neck) and three (angiography head thorax and abdomen) | Two phases with the arms positioned ventral on a pillow to the torso throughout the entire CT – native HN and Split bolus | - |
| L. Simma et al. | Three phases - native cranial, angiography of the neck, TAP (two bolus injection) | Split-bolus: unenhanced H, split-bolus NTAP | - |
| **Position of the arms** | | | |
| C. M. Heyer et al. | Unenhanced HN with arms by body, gantry tilt at skull base (H) or 2nd vertebral body (N), serial scan (H) and spiral scan (N) / enhanced TAP with arms over head, no gantry tilt, spiral scan | Unenhanced HN without gantry, arms by side, spiral scan / enhanced TAP without gantry tilt, arms by side, spiral scan | - |
| **J**. Bayer et al. | Sequential scan of head and cervical spine with arms beside the trunk, then repositioning of the arms above head and acquisition of combined contrast enhanced scan of TAP | Sequential scan of head and cervical spine with arms beside the trunk, no reposition during acquisition of combined contrast enhanced scan of TAP | Sequential scan of head and cervical spine with arms beside the trunk, repositioning of one arm above head and acquisition of combined contrast enhanced scan of TAP |
| C. Karlo et al. | Native HN with arms alongside body, enhanced TAP with arms above head (Arterial and venous phases) | Same protocol but both arms alongside the body, | Same protocol but arms ventrally over a large pillow (height of the pillow=15 cm) ventrally on the body at the level of the lower chest. |
| A. Harrieder et al. | 64-row WBCT, unenhanced H, enhanced NT, enhanced AP (arms crossed during abdominal) | 4-row WBCT, unenhanced H, enhanced NT, enhanced AP (arms upwards during thoraco-abdominal) | - |
| **AEC and Arms position** | | | |
| S. U. Reske et al. | Unenhanced HN and one contrast-enhanced venous body scan without dose modulation and without gantry tilting. Arms close to the body and the complete WBCT scan was planned based on a long whole-body scout. | Unenhanced HN, WBCT venous phase with AEC, with the arms close to the body | Short scout, Unenhanced HN, then patients arms positioned overhead with new scout acquired for appropriate AEC, contrast-enhanced WB with arms over head |
| **Single pass (vs segmented)** | | | |
| E. Fanucci et al. | single-pass continuous wholebody acquisition (from vertex to pubic symphysis) without gantry angulations or arm elevation | conventional segmented acquisition with the scanning of body segment, individually, with overlap zones between body segments and also adjusting gantry angulations for the cranium and suitable FOV for facial bone, but without arm elevation |  |
| T. Ptak et al. | Single-pass continuous WB acquisition | Segmented scanning of body segments |  |
| A. Sedlic et al. | Conventional: head and neck (non-contrast), arterial chest, portovenous CT abdomen pelvis after 54s, arterial phase from arch to Circle of Willis. Delayed abdomen to pelvis. Arms down when doing head/neck, thorax arms up for abdo/pelv. Split bolus for contrast protocol | Single acquisition dual-source WBCT - Rapid imaging protocol in trauma (RIPIT), both arms up. Triphasic injection protocol (non contrast CT head, WB angio, Portovenous abdopelvis) | - |
| *Table 1 – Protocols used, with the one that achieved lower-dose underlined. WB : whole body; TAP: Thoracic, abdominal and pelvis scan; H: head; NT : neck and thorax; AP abdominal and pelvis scan* | | | |

| **Table 2 - Dose calculation method** | |
| --- | --- |
| **AUTHOR** | **Effective dose method of calculation** |
| **Iterative reconstruction** | |
| U. Grupp et al | DLP*conversion coefficient 𝑘 of 0.017mSv/mGy⋅cm (Yeguiayan 2012, Impact of whole-body computed tomography on mortality and surgical management of severe blunt trauma) |
| J. Kahn et al. | Not done |
| L. L. Geyer et al. | DLP*coefficient 0.0023 for head, 0.017 thorax and 0.015 for abdomen (Monte Carlo simulation IRCP 60 organ. European Commission. European Guidelines on Quality Criteria for Computed Tomography 1999) |
| J. Kahn, U et al. | DLP*conversion coefficient IRCP with corrected dose estimates for the body region according to the AAPM report 204 (Deak 2010, Multisection CT protocols: sex- and age-specific conversion factors used to determine effective dose from dose-length product) |
| Z. Alagic et al. | Method not explained |
| D. Stengel et al. | Not done |
| **Split bolus** | |
| G. Yaniv et al | DLP * conversion factor, not given probably ICRP 103 (reference to two articles Huda 2008, Converting dose length product to effective dose at CT and Christner JA 2010, Estimating effective dose for CT using dose length product compared with using organ doses) |
| L. F. Beenen et al. | Method not explained |
| V. Leung et al. | Method not explained |
| W. Hakim et al. | DLP*conventional conversion factor (Christner Estimating effective dose for CT using dose length product compared with using organ doses: consequences of adopting International Commission on Radiological Protection publication 103 or dual-energy scanning 2010) |
| A. H. Elmokadem et al. | DLP*normalization coefficient 0.0031 for HS, 0.014 for chest, 0.015 for abdo and pelvis (American Association of Physicists in Medicine. Report No. 096—The Measurement, Reporting, and Management of Radiation Dose in CT 2008 |
| C. A. Ordoñez et al | Method not explained |
| S Studer et al. | Multiplying conversion factors times the DLP for each examination (Bongartz G, Golding SJ, Jurik AG. European guidelines for multislice computed tomography: appendix C. Funded by the European Commission. 2004. http://www.msct.eu/PDF_FILES/EC%20CA%20Report%20D5%20-%20Dosimetry.pdf Accessed ) |
| L. Simma et al. | National Cancer Institute dosimetry system for CT - Monte Carlo + phantoms Lee 2015 NCICT: a computational solution to estimate organ doses for paediatric and adult patients undergoing CT scans |
| **Position of the arms** | |
| C. M. Heyer et al. | Method not explained |
| J. Bayer et al. | DLP*k = 0.017 mSv mGy-1 cm-1 (European Guidelines on Quality Criteria for Computed Tomography. Luxembourg) |
| C. Karlo et al. | DLP*conversion coefficient k= 0.0145 ( mean from thoracic 0.014 and abdominal 0.015). Menzel 2000, European guidelines |
| A. Harrieder et al. | DLP*coefficient 0.0023 for head, 0.017 thorax and 0.015 for abdomen (Monte Caroli simulation IRCP 60 organ. European Commission. European Guidelines on Quality Criteria for Computed Tomography 1999) |
| **AEC and arms position** | |
| S. U. Reske et al. | Method not explained |
| **Single pass (vs segmented)** | |
| T. Ptak et al. | Not done |
| E. Fanucci et al. | Not done |
| A. Sedlic et al. | DLP*coefficient, not given (Mayo 2009, Radiation dose in cardiac CT and Huber-Wagner 2009 Working Group on Polytrauma of the German Trauma Society |
| Method used for each radiation dose with reference. * multiplication | |

| **Table 3 - OVID Medline search string** | | |
| --- | --- | --- |
| #18 | 13 and 14 and 15 | 71 |
| #17 | 13 and 15 | 227 |
| #16 | 13 and 14 | 79 |
| #15 | humans.mp. or Humans/ | 21964251 |
| #14 | Emergency Service, Hospital/ or emergen*.mp. or Emergency Medical Services/ or Emergencies/ | 670735 |
| #13 | 11 and 12 | 4153 |
| #12 | 3 and 9 and 10 | 11774 |
| #11 | 7 or 8 | 7516835 |
| #10 | 4 or 5 or 6 | 38092 |
| #9 | 1 or 2 | 7722837 |
| #8 | Trauma* or Wound* or Injur* or Shock* or Emergen* or "Multi* trauma*" or "Multi* injur*" or "Severe injur*" or "Severe trauma*" or Polytrauma* or "Major trauma*" or "Major injur*" | 7213083 |
| #7 | trauma.mp. or exp "Wounds and Injuries"/ | 1203312 |
| #6 | "multi-slice spiral computed tomography" or "multi-detector computer tomography" or "multi-slice spiral CT" or "multi-detector CT" or "MSCT" or "MDCT" | 29823 |
| #5 | whole*body or total*body or full*body or WBCT or FBCT or TBCT or Pan*scan or pan*CT or pan*CAT or "pan computed tomography" | 2786 |
| #4 | exp Whole Body Imaging/ | 5696 |
| #3 | ((low* or reduc* or diminish* or small* or attenuat* or inferior or lesse* or decrease*) and (dos* or radiation*)) or ldct* or rdct* | 5044024 |
| #2 | exp Tomography, Spiral Computed/ or exp Tomography, X-Ray Computed/ or exp Image Processing, Computer-Assisted/ or CT.mp. | 948362 |
| #1 | imag* or scan* or "computed tomography" or "computerized tomography" | 7578653 |

| **Table 4 - Quantitative evaluation** | | | | |
| --- | --- | --- | --- | --- |
| **AUTHOR** | **Quantitative analysis of image** | **Conventional** | **Low dose** | **Statistical sig** |
| **Iterative reconstruction** | | | | |
| U. Grupp et al.. | Image noise= standard deviation (SD) of attenuation value. Lower is better | | | |
|  | Supracarinal trachea (HU), | 3.9 ± 0.7 HU | 3.3 ± 0.6 HU | NS |
| J. Kahn, U et al. | SNR and CNR. Higher is better. Values in other organs and noise measured, but not all results given | | | |
|  | SNR mean | 114.70 ± 33.38 | 127.03 ± 59.56 | NS |
|  | CNR Liver | 43.72 ± 11.18 | 49.67 ± 13.33 | S |
| J. Kahn et al. | SNR, CNR using attenuation values (SI) in Hounsfield units (HU) and the standard deviation measured in oval regions of interest (ROI) of at least 1 cm2 size in body regions that are often affected by trauma | | | |
|  | SNR liver | 15.7 ± 4.2 | 22.5 ± 4.8 | S |
|  | CNR liver parenchyma-fat tissue | 31.7 ± 8.5 | 40.5 ± 7.6 | S |
|  | CNR bone-fat tiusse | 49.6 ± 15.3 | 56.4 ± 13.9 | NS |
|  | HU aorta | 219.9 ± 50.9 | 271.0 ± 69.6 | S |
|  | HU portal vein | 137.5 ± 25.1 | 213.4 ± 41.0 | S |
| Z. Alagic et al. | Mean Houndsfield unit (HU) values with standard deviation (SD) and signal-to-noise ratios (SNRs) during native scull, native cervical spine, and portal venous phase. Protocol convent = natif + vp. Protocol LD = natif + angio + vp | | | |
|  | HU Abdominal aorta mean HU | 39 | 19 | S |
|  | HU Liver (VIII) mean HU | 34 | 18 | S |
|  | SNR Abdominal aorta | 4 | 8 | S |
|  | SNR Liver (VIII) | 3 | 7 | S |
| D. Stengel et al. | CNR Reference region was placed in muscle tissue adjacent to the individual landmark | | | |
|  | CNR carotid artery | 11.7 | 28.9 | S |
|  | CNR aortic arch | 15.1 | 17.8 | S |
|  | CNR liver | 2.0 | 2.5 | S |
|  | CNR aorta | 11.6 | 13.9 | S |
| **Split bolus** | | | | |
| G. Yaniv et al | Attenuations in Hounsfield units readings of blood vessels and parenchymatous organs were measured using the PACS system. The radiodensity was measured by placing the region of interest (ROI) on the selected area. | | | |
|  | Ascending aorta | 285 ± 10.5 | 217 ± 15.84 | S |
|  | Aorta above kidneys | 125.6 ± 3.15 | 208.8 ± 15 | S |
|  | IVC | 111.5 ± 2.7 | 147.6 ± 6 | S |
|  | Liver | 96.2 ± 2.2 | 109.9 ± 5.6 | S |
| L. F. Beenen et al. | Hounsfield Units (HU) attenuation determined by setting a region of interest (ROI) half of the vessel caliber for the aortic arch, abdominal aorta, and the portal vein were registered, as well as in the parenchyma of the liver, spleen, and renal cortex using a 1 cm ROI. | | | |
|  | Aortic arch | 177.8 HU | 275.9 HU | S |
|  | Abdominal aorta | 172.8 HU | 241.4 HU | S |
|  | Portal vein | 140.3 HU | 155.5 HU | NS |
|  | Liver | 82.9 HU | 78 HU | NS |
| V. Leung et al. | Vascular enhancement was measured using a region of interest (ROI) tool that provided the average radiodensity (in Hounsfield units) of the encircled region. Aortic radiodensity was measured at the level of the coeliac axis and the radiodensity of the portal vein was measured just proximal to its bifurcation | | | |
|  | Abdominal aorta | 326.2 ± 22.4 HU | 269.8 ± 18.8 HU | S |
|  | Portal vein | 167.4 ± 8.7 HU | 246.1 ± 16.3 HU | S |
| W. Hakim et al. | The arterial, venous and parenchymal attenuation profiles were generated using 1-cm regions of interest (ROIs) in specific locations. The ROI was carefully positioned in the centre of each blood vessel for vascular opacification. For the conventional protocol, arterial ROIs were measured in arterial phase imaging and venous ROIs were measured in PV phase imaging. In the solid organs, a 1-cm ROI was placed in parenchyma free of injury, vessels and artefacts where possible. Renal cortex and medulla were both included in a single ROI. Mean and standard deviation enhancement values (Hounsfield units) were calculated for each scan protocol. | | | |
|  | Ascending aorta | 259 ± 17 | 218 ± 17 | S |
|  | Infrarenal aorta | 305 ± 21 | 256 ± 29 | S |
|  | IVC | 124 ± 28 | 157 ± 35 | S |
|  | Liver | 101 ± 23 | 113 ± 27 | NS |
|  | Spleen | 103 ± 24 | 165 ± 25 | S |
| A. H. Elmokadem et al. | The contrast enhancement was measured using 1-cm regions of interest (ROIs). The ROI was carefully positioned in the center of each blood vessel for optimum enhancement value. In the solid organs, the ROI was placed in parenchyma free of injury, vessels, and artifacts where possible. | | | |
|  | Ascending aorta | 292.8 ± 47.4 | 294.6 ± 78.6 | NS |
|  | IVC suprarenal | 154.3 ± 17.7 | 166.0 ± 41.1 | NS |
|  | Liver | 124.5 ± 14.0 | 115.7 ± 17.8 | S |
| **Position of the arms** | | | | |
| C. Karlo et al. | ROI with a diameter of 1 cm in the right lobe of the liver at the level of the right portal vein in all patients (i.e., liver segment VI or VII), excluding focal liver lesions and major hepatic vessels, as previously shown. The mean of the standard deviation of the CT number measurement in Hounsfield Units (HU) determined the image noise. Lower is better | | | |
|  | Mean image noise | 20 ± 5 HU | 18±4 HU | S |
| **AEC and Arms position** | | | | |
| S. U. Reske et al. | The objective image quality was defined as image noise (= IN = standard deviation of Hounsfield units in a ROI). | | | |
|  | Aorta upper abdomen | 18.6 ± 5.3 | 14.1 ± 2.2 | S |
|  | Liver VII | 21 ± 6.9 | 14.6 ± 1.8 | S |
| **Single pass (vs segmented)** | | | | |
| A. Sedlic et al. | Standard deviation of the attenuation of the aorta at the left renal artery origin on the portovenous phase and by measuring image noise, defined as standard deviation of the attenuation measured within the thoracic aorta, immediately distal to the left subclavian artery origin and measured during the arterial phase scan of the thorax. | | | |
|  | Mean noise level | 14.9 ± 5.5 HU | 13.3 ± 4.0 HU | NS |
| Results of quantitative evaluation in each study. Description and parameter used to describe given for each article. S : result is statistically significative. NS: result is not statistical significative. | | | | |
